# The prevalence of alcohol and illicit drug use among injured patients presenting to the emergency department of a national hospital in Tanzania: a prospective cohort study

**DOI:** 10.1186/s12873-019-0222-9

**Published:** 2019-01-24

**Authors:** Müller M. Mundenga, Hendry R. Sawe, Michael S. Runyon, Victor G. Mwafongo, Juma A. Mfinanga, Brittany L. Murray

**Affiliations:** 1 Department of Emergency Medicine, Muhimbili University of Health and Allied Sciences, Dar es Salaam, Tanzania; 2 Heal Africa Hospital, Goma, Democratic Republic of Congo; 3 Department of Emergency Medicine, Muhimbili National Hospital, Dar es Salaam, Tanzania; 4 Department of Emergency Medicine, Carolinas Medical Center, Charlotte, NC USA; 5 Division of Pediatric \Emergency Medicine, Emory University School of Medicine/Children’s Healthcare of Atlanta, Atlanta, GA USA

**Keywords:** Alcohol, Illicit drugs, Injured patients, Road traffic injury, Emergency medicine

## Abstract

**Background:**

Alcohol and illicit drugs have been found to be major contributing factors leading to severe injuries in a variety of settings. In Tanzania, the use of these substances among injured patients has not been studied. We investigated the prevalence of positive tests for alcohol and illicit drug use among injured patients presenting to the emergency medicine department (ED) of Muhimbili National Hospital (MNH).

**Methods:**

This was a prospective cohort study of a consecutive sample of patients > 18 years of age presenting to the ED-MNH with injury related complaints in October and November 2015. A structured data sheet was used to record demographic information, mechanism of injury, clinical presentation, alcohol and illicit drug test results, and ED disposition. Alcohol levels and illicit drug use were tested by breathalyser device or swab stick alcohol test and multidrug urine panel, respectively. Patients were followed up for 24 h and 30 days using medical chart reviews and phone calls. Descriptive statistics and relative risk were used to describe the results.

**Results:**

We screened 1011 patients and we enrolled all 143 (14.1%) patients who met inclusion criteria. 123 (86.0%) were male, the median age was 30 years (IQR: 23–36 years). The most frequent mechanism of injury was road traffic accidents (84.6%). 67/143 (46.9%) patients tested positive for alcohol and 44/122 (36.1%) patients tested positive for drugs. 29 (26.1%) tested positive for alcohol and drugs. The most frequently detected illicit drug was marijuana in 30/122 (24.5%) injured patients. 23/53 (43.4%) patients with positive alcohol testing self-reported alcohol use. 3/25 patients with positive illicit drug tests who were able to provide self-reports, self-reported drug use. At 30-day followup, 43 (64.2%) injured patients who tested positive for alcohol had undergone major surgery, 6 (9.0%) had died, and 36 (53.7%) had not yet returned to their baseline.

**Conclusions:**

The prevalence of alcohol and illicit drugs is very high in patients presenting to the ED-MNH with injury. Further studies are needed to generalise the results in Tanzania. Public health initiatives to decrease drinking and/or illicit drug use and driving should be implemented.

**Electronic supplementary material:**

The online version of this article (10.1186/s12873-019-0222-9) contains supplementary material, which is available to authorized users.

## Background

In high-income countries (HIC), hospital-based emergency department (ED) studies have demonstrated a clear link between alcohol and illicit drug use and the occurrence of injuries, as well as injury-related deaths [1]. In low- and middle-income countries (LMIC) little is known about alcohol and drug use among injured patients even though the World Health Organization (WHO) estimates that 90% of injury-related deaths worldwide occur in LMIC [2, 3]. The few studies that have been done in sub-Saharan Africa to illustrate the prevalence of alcohol and illicit drug use among injured patients show that alcohol is the most commonly described substance [4–6].

Although many studies conducted in both HIC and LMIC have used patient report to screen for alcohol and illicit drug use in injured patients, analytical testing has been shown to be more accurate, specifies the substances used, allows this testing in patients with altered mental status, and avoids underestimation of alcohol and drug use [7–9].

In Africa, the majority of studies on substance use in injured patients rely on patient report of alcohol and illicit drug use, with the exception of few studies conducted in the Ivory Coast, Ghana, and South Africa which used objective testing [10–12]. In Tanzania, most studies examining alcohol and illicit drug use have been done in non-injured patients [13, 14]. Unfortunately, none of those studies have used objective testing to screen for alcohol and illicit drugs use, and none of them focused on injured patients presenting to an ED of a tertiary level facility. As severely injured patients are referred to tertiary level facilities in Tanzania, this is an important distinction.

The aim of this study was to determine the prevalence of injured patients with a positive test for alcohol and/or illicit drug use, to characterize the various substances of abuse found, to compare the patient report of substance abuse with the results of analytic testing, and to determine the 24-h and 30-day morbidity and mortality rates of injured patients with positive tests for alcohol and/or illicit drugs. This information will provide an improved understanding of the scope of this problem in Tanzania and will help to guide future research and public health intervention strategies that may benefit patients in our region.

## Methods

### Study design

This is a prospective descriptive cohort study of a consecutive sample of 143 patients aged 18 years and above presenting to the ED-MHH, Dar es Salaam, Tanzania within 12 h of sustaining an injury between 17th October 2015 and 4th November 2015.

### Study setting and population

This study was conducted at the Emergency Department of Muhimbili National Hospital (ED-MNH). MNH is the national hospital of Tanzania. It is located in Ilala district, Dar es Salaam, Tanzania. MNH has a capacity of 1500 beds. MNH is the highest level of medical care available in the government system in Tanzania, and thus severely injured patients from across the country are referred to MNH. When arriving at MNH, these patients are first evaluated in the ED-MNH. The ED-MNH is open 24 h every day and sees an average of 60,000 patients per year, including approximately 1500 trauma patients per month. It has 3 adult resuscitation rooms and one paediatric resuscitation room. The ED was built in 2009 and opened to patients in January 2010; it is the only recognized public ED in Tanzania. The staff of the ED-MNH is composed of emergency medicine trained faculty, residents, registrars, interns, and nurses. It is a teaching department with a formal residency program which was started in 2010 [15]. Drug and alcohol testing is currently not routinely available at government hospitals in Tanzania. This testing is only performed in special toxicological or suspicious legal cases by the chief chemist of the Government. Our study population included all injured patients aged 18 years and above who arrived at the ED-MNH within 12 h of their injury.

### Study protocol

Patient testing and enrollment was completed by the principal investigator and a trained research assistant. All patients arriving at the ED-MNH were screened for eligibility. For patients who met inclusion criteria, we obtained written informed consent. This consent was obtained from the patient if they had capacity, or from the appropriate proxy/relative if the patient had altered mental status. A structured data collection form was used to record age, sex, location of the injury, mechanism of the injury, severity of the injury, patient and relative report of alcohol and illicit drug use, and nurse, and physician presumption of alcohol and drug use.

Blood alcohol concentration was measured by using a Breathalyzer (BACtrack Select S80 Breathalyzer) for those able to properly use the breathalyser. For patients unable to properly use the breathalyser, saliva swab sticks (*Instant Alcohol Saliva Test Strips/W53-S*) were used to measure the level of alcohol. The*RapidCHECK*® *5 Multi-Drug* Panel Test, was used to identify illicit drugs using urine samples. It screened for the presence of opioids, cocaine, marijuana, amphetamines, and benzodiazepines.

### Key outcome measures

The primary outcomes were: 1) the prevalence of injured patients with a positive test for alcohol/ illicit drugs at the time of presentation to the ED-MNH; 2) the characteristics of the various substances of abuse among injured patients with a positive test for alcohol and/or illicit drug use at the time of presentation to the ED-MNH; 3) the comparison of patient report, relative report and healthcare provider suspicion of alcohol and/or illicit drug use with the results of objective testing for alcohol and illicit drug use among injured patients presenting to the ED-MNH.

The secondary outcomes were 24-h and 30-day morbidity and mortality rates of injured patients with a positive test for alcohol and/or illicit drugs presenting to the ED-MNH.

All patients were followed up for 30 days and admission to the hospital, need for surgery, 24-h mortality, 30-day mortality, and 30-day return to baseline were recorded.

### Data analysis

Data was collected on hand-written data collection forms and then entered into Microsoft Excel (Microsoft Corporation, Redmond, WA) and analysed with Microsoft Excel. Data was summarized in tables using descriptive statistics, percentages, inter quartile ranges, sensitivity and specificity, positive and negative predictive value, relative risk, and 95% confidence intervals were used as appropriate.

## Results

### Study population demographics

**Fig. 1:**
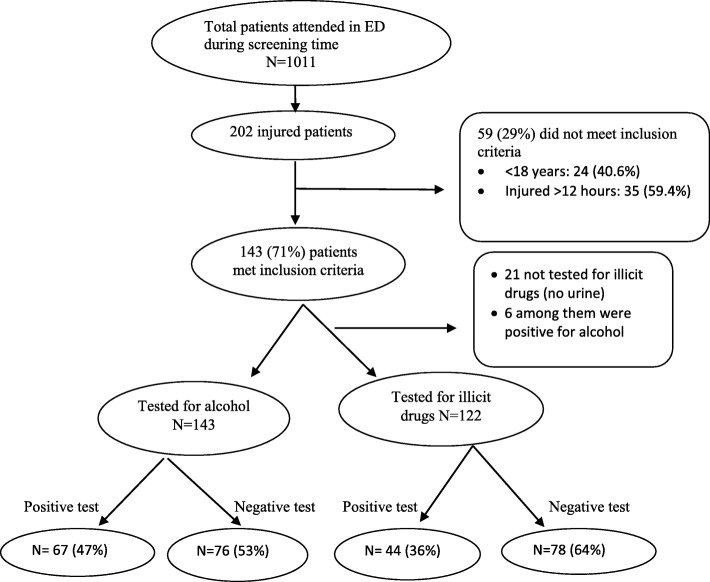
Study flow chart: Inclusion criteria, Alcohol/illicit drug abuse testing

During the study period, a total of 1011 adult patients were attended at the ED-MNH, of which 202 had trauma related complaints. Out of 202 patients with trauma, 143 (70.8%) met the inclusion criteria for the study (Fig. 1). All 143 eligible patients were enrolled. 86.0% (*n* = 123) of patients were male, and their median age was 30 years (IQR 23–36). All 143 patients enrolled were tested for alcohol. Because we were unable to obtain urine from 21 patients during their time in the ED, we tested only 122/143 (85.3%) patients for illicit drugs. 121 (84.6%) of patients were injured on the road, followed by 14 (9.8%) at home, and other locations (5.6%) as shown in Table 1. 80.4% (*n* = 115) of patients were referred from peripheral health centers, 10.4% (*n* = 15) were self-referred, and 9.1% (*n* = 13) were brought by the police (Table 1).

**Table 1:** Study population demographics

	Total enrolled in the study *N* = 143 *n* (%) [CI]	Injured patients tested for both alcohol and illicit drugs *N* = 122 *n* (%) [CI]	Injured patients tested only for alcohol *N* = 21 *n* (%) [CI]
Gender			
Female	20 (14%) [8.4–19.6%]	16 (13%) [7.1–18.9%]	4 (19%) [2.3 to 35.7%]
Male	123 (86%) [80.4–91.6%]	106 (87%) [81.1–92.9%]	17 (81%) [64 to 97.7%]
Median age			
Age Median	30	30	33
(IQR) years	23–36	23–37	26–35
Place of injuries			
Road	121 (84.6%) [77.6–90.1%]	92 (75.4%) [67.8–83%]	16 (76%) [58 to 94%]
Home	14 (9.6%) [4.8–14.4%]	11 (9%) [4–14%]	3 (14.3%) [3.0 to 36.3%]
Work site	6 (4%) [0.8–7.2%]	5 (4%) [1.4–7.4%]	1 (4.%) [0.1 to 23.8%]
Public place	1 (0.7%) [0.6–2%]	0	0
Unreported	1 (0.7%) [0.6–2%]	1 (0.8%) [0.7–2.3%]	0
Referral status			
Referred	115 (80%) [73.5–86%]	105 (86%) [79.9%-92.1]	10 (47%) [25.7–68.3%]
Self referral	15 (11%) [5.9–16.1%]	8 (6.5%) [2.2–10%]	7 (33.3%) [13.2–53.4%
Police	13 (9%) [4.4–13.6%]	9 (7.3%) [2.7–11.9%]	4 (19%) [2.3–35.7%]

### Results of alcohol and illicit drug testing among injured patients

67 (46.9, 95% CI: 39.3–54.7%) patients tested positive for alcohol. Among the 122 patients who were tested for illicit drugs, 44 (36.0, 95% CI: 27.2–44.8%) tested positive. Out of the 122 injured patients who were tested for both alcohol and illicit drugs, 29 (23.7, 95% CI: 16.5–32.3%) tested positive for both alcohol and illicit drugs, 46 (37.7, 95% CI: 29.1–46.9%) tested negative for both alcohol and illicit drugs, and 82 (57.3, 95% CI: 49.2 to 69.1%) patients tested positive for either alcohol or illicit drugs. (Table 2)

**Table 2:** Results of alcohol and illicit drug testing among injured patients

Test	Count (%) [CI]
Alcohol positive (*n* = 143)*	67 (47%) [39.3 to 54.7%]
Illicit drugs positive (*n* = 122)**	44 (36%) [27.2 to 44.8%]
Alcohol and illicit drugs positive (*n* = 122)	29 (24%) [16.5 to 31.5%]
Either alcohol or illicit drugs positive (*n* = 143)	82 (57%) [49.2 to 69.1%]
Both alcohol and illicit drugs negative (*n* = 143)	46 (32%) [24.6 to 40.5%]

*This includes the patients who tested positive for alcohol alone, and those who tested positive for alcohol and illicit drugs

**This includes the patients who tested positive for illicit drugs alone, and those who tested positive for alcohol and illicit drugs

Among the 67 patients who tested positive for alcohol, 42 (62.7%) had blood alcohol concentrations between 0.01 and 0.04%; 9 (13.4%) had blood alcohol concentrations between 0.05 and 0.09%, and 16 (23.9%) had blood alcohol concentrations greater than 0.1%. Marijuana was the most common illicit drug detected and was found in 24.5% (*n* = 30) of patients, followed by opioids 11.5% (*n* = 14), benzodiazepines 9.8% (*n* = 12), and amphetamines 2.5% (*n* = 3). Cocaine was not detected in any patient. Among the 122 patients tested for both alcohol and illicit drugs, 29 (23.8%) patients tested positive for more than one substance of abuse. Of those patients, 22 (18.0%) tested positive for two substances, 6 (4.9%) tested positive for three substances, and 1 (0.8%) tested positive for four substances.

### History and presumption of alcohol and illicit drugs among injured patients versus the results of analytic alcohol and illicit drug testing

Among the 143 enrolled in the study, 33 patients were not able to answer questions because of altered mental status. Among 53 patients with positive alcohol test that could answer the question, 23 (43%) reported the use of alcohol and among the 25 patients with positive illicit drug test that could answer the question, 3 (12%) reported illicit drug use. Among the 61 patients with positive tests for alcohol whose relatives were available for questioning, 34 (56%) of relatives reported that the patient used alcohol prior to the injury and among the 44 patients with positive test for illicit drug use whose relatives were available, 9 (82%) of the relatives reported the patient had used illicit drugs. Thus, when the patient reported that they had used alcohol, the relative risk of a positive test result was 2.4 (95% CI: 1.7 to 3.3). However, when the patient denied alcohol use, the test was positive in 30/83 (36%) cases. Likewise, when the patient reported that they had used illicit drugs, the relative risk of a positive test result was 3.6 (95% CI: 1.4 to 6.0). However, when the patient denied illicit drug use, the test was positive in 22/106 (21%) cases. Similarly, when a relative reported that the patient had used alcohol, the relative risk of a positive test result was 3.2 (95% CI: 2.3 to 4.6). However, when a relative reported no alcohol use, the test was positive in 27/103 (26%) cases. Finally, when a relative reported that the patient had used illicit drugs, the relative risk of a positive test result was 2.6 (95% CI: 1.6 to 3.7). However, when the relative reports no Illicit drug use, the test was positive in 35/111 (32%) cases (Table 3).

**Table 3:** History and presumption of alcohol/illicit drug use versus test results

			Alcohol test result			Illicit drugs test result	
History of use		History of alcohol	Positive *n* = 53	Negative *N* = 57	History of illicit drugs	Positive *n* = 25	Negative *n* = 89
	Patients	Yes	23	4	Yes (*n* = 4)	3	1
		No	30	53	No (*n* = 106)	22	84
		History of alcohol	Positive *n* = 61	Negative *n* = 82	History of illicit drugs	Positive *n* = 44	Negative *n* = 78
	Relatives	Yes	34	6	Yes	9	2
		No	27	76	No	35	76
Presumption of use		Presumption of alcohol	Positive *n* = 45	Negative *n* = 98	Presumption of illicit drugs	Positive *n* = 26	Negative *n* = 98
	Relatives	Yes	10	38	Yes	8	4
		No	35	60	No	18	92
		History of alcohol	Positive *n* = 52	Negative *n* = 91	History of illicit drugs	Positive *n* = 22	Negative *n* = 100
	Nurses	Yes	43	18	Yes	1	6
		No	9	73	No	21	94
		History of alcohol	Positive *n* = 124	Negative *n* = 101	History of illicit drugs	Positive *n* = 27	Negative *n* = 95
	Doctors	Yes	44	18	Yes	2	4
		No	8	73	No	25	91

Among the 52 patients with positive tests for alcohol, in 43 (83%) patients, the nurse presumed the patient had used alcohol. The sensitivity and specificity for the nurses’ presumption of alcohol use as a predictor of the test result were 83% (95%CI: 70 to 92%) and 80% (95%CI: 71 to 88%), respectively. Among the 22 patients with positive illicit drug tests, in 1 (5%) patient, the nurse presumed the patient’s use of illicit drugs. The sensitivity and specificity for the nurses’ presumption of illicit drug use as a predictor of the test result were 5% (95%CI: 0 to 23%) and 94% (95%CI: 87 to 98%), respectively. Among the 124 patients with a positive test for alcohol, physicians presumed alcohol use in 44 (85%) patients. The sensitivity and specificity for the doctors’ presumption of alcohol use as a predictor of the test result were 85% (95%CI: 72 to 93%) and 80% (95%CI: 71 to 88%), respectively. Among the 27 patients with positive test for illicit drug abuse, physicians presumed 2 (7%) of the patients had used illicit drugs. The sensitivity and specificity for physician presumption of illicit drug use as a predictor of the test result were 7% (95%CI: 1 to 24%) and 96% (95%CI: 90 to 99%), respectively.

### 24-h and 30-days morbidity and mortality

At 24 h and 30 days, the patients with positive alcohol tests were statistically more likely to have required major surgery than the patients with negative alcohol tests (RR 1.42, 95%CI 1.09 to 1.85); the patients with positive tests for illicit drugs were also statistically more likely to get major surgery than the patients with negative tests for illicit drugs (RR: 1.59, 95% CI: 1.23 to 2.06).

Although the results did not reach statistical significance, patients who tested positive for multiple substances were more likely to get major surgery than the patients who were positive for a single substance ingestion (RR: 1.2; 95% CI: 0.73 to 1.05); patients with positive isolated alcohol test were less likely to get major surgery than the patients who tested positive for only drugs (RR: 0.93, 95% CI: 0.64 to 1.35); the patients with positive test for alcohol were more likely to have a prolonged return to baseline than those who were negative for alcohol (RR: 1.19, 95% CI: 0.88 to 1.60); the patients with positive test for illicit drug were more likely to have a prolonged return to the baseline than those who tested negative for illicit drugs (RR: 1.33, 95% CI: 0.97 to 1.83); the patients with positive tests for multiple substance ingestions were more likely to have a prolonged return to the baseline compared to those who were tested positive for one substances abuse (RR: 1.12; 95% CI: 0.81 to 1.56); the patients with positive test for isolated alcohol use were more likely to have prolonged return to baseline compare to those with isolated illicit drugs use (RR: 1.1, 3, 95% CI: 0.66 to 194); the patients who tested positive for alcohol were more likely to die than those who tested negative for alcohol (RR: 1.70, 95% CI: 0.50 to 5.77); the patients with positive testing for illicit drugs were more likely to die than those who tested negative for illicit drugs (RR: 3.54, 95% CI: 0.67 to 18.5); the patients who tested positive for multiple substances were more likely to die than those with single substances ingestion (RR:1.82, 95% CI: 0.34 to 9.57); the patients with positive testing for isolated alcohol use were less likely to die than those who tested positive for isolated drug use (Additional file 1) (RR: 0.39, 95% CI: 0.02 to 5.91) (Table 4).

**Table 4:** 24-h and 30-day outcomes: relative risks of major surgery, return to the baseline, and death among injured patients testing positive for alcohol, illicit drugs, or a combination of both

Categories	Major surgery		Not returned to baseline		Death	
	RR	95% CI	RR	95% CI	RR	95% CI
Positive for alcohol vs negative for alcohol	1.42	1.09 to 1.85	1.19	0.88 to 1.60	1.70	0.50 to 5.77
Positive for illicit drugs vs negative for illicit drugs	1.59	1.23 to 2.06	1.33	0.97 to 1.83	3.54	0.67 to 18.5
Positive for multiple substances ingestion vs single substance ingestion	0.87	0.73 to 1.05	1.12	0.81 to 1.56	1.82	0.34 to 9.57
Positive for isolated alcohol vs isolated drug use	0.93	0.64 to 1.35	1.13	0.66 to 1.94	0.39	0.02 to 5.91

RR relative risk; 95%CI: 95% confidence interval

## Discussion

The demographic characteristics of our study population showed a male predominance 123 (86.0%), with a median age of 30 years (23–36 years) and 85% of patients enrolled in this study were the victims of road traffic injury (RTI). This is a similar to the reported trauma burden facing many LMICs, in which, morbidity and mortality from RTIs have taken a sharp rise over the last decade due to rapid urbanization and population growth [16–19]. In this cohort, most of the victims were drivers of motorcycles, famously known as “boda-bodas”, which are local motorcycle taxis used to ferry patients to different parts of the main city. This is a concerning revelation that needs urgent policy and law enforcement to protect the drivers, passengers and pedestrians.

We found that 47% of the injured patients presenting to our ED tested positive for alcohol, 36% tested positive for the use of illicit drugs, and nearly 29% tested positive for both alcohol and illicit drugs. These are very high rates of substance abuse use compared to most of the studies we came across, including the global report on substance abuse in 2014 which reported the prevalence of alcohol and illicit drug use range respectively between 6 to 45% and 2 to 25% [14, 20–22]. In our study, alcohol was the most frequent substance found amongst injured patients (46.9%), followed by marijuana (24.5%), opioids (11.4%), benzodiazepines (9.8%) and amphetamines (2.4%). Marijuana was most prevalent in younger patients (median age of 26 years). Many studies on alcohol and illicit drug use conducted worldwide reported similar results as ours in terms of which substances are most frequently detected in injured patients [22–24]. The high proportion of young males using drugs and alcohol and sustaining injuries in our study is particularly concerning as this is the age group that is expected to be economically productive, and often represent the breadwinner in Tanzanian households [13, 14]. The striking number of road traffic injuries and substance use could be due to the increased availability of low-cost motorization (motorcycles), poor road infrastructure, poor enforcement of traffic laws, low rates of education, high rate of unemployment, and poor socioeconomic status in combination with relatively cheap alcohol and drugs and poor law enforcement of regulations regarding drinking and driving [12, 25–27]. This is clearly an issue that deserves additional research, and these findings begin to provide the opportunity for stratification of behavior change campaigns and public health efforts.

In this study, the results of the substances abuse screening show that 57% of the patients denied the use of alcohol and 88% denied the use of illicit drug use before the injury while the objective testing showed that they were positive. Furthermore, the presumption of patient substance abuse use by their relatives was only 12% positive for alcohol and 31% positive for illicit drugs. The nurses and doctors’ patient presumption of substances abuse use was better for alcohol than for illicit drug use. This highlights the importance of analytic testing to define this issue. Although many studies worldwide have used patient’s verbal report or a questionnaire to screen for alcohol and illicit drug use in injured patients, our study and other studies, mostly conducted in HIC, have demonstrated that analytical testing is more accurate, gives the proof of substances abuse consumption, gives information when patients are unable to, and avoids under estimation of drug and alcohol use [7–9]. One of the biggest limitations of analytic testing in our setting is the high cost of alcohol and drug testing, and this is true in most LMICs [28].

At 24-h and 30-day followup we found that injured patients who tested positive for substance abuse were more likely to require major surgery than those who tested negative. The outcomes and relative risk findings above suggest that substance abuse contributes to more severe injuries leading to surgery, and that these trauma patients had high rates of prolonged return to the normal activities, and of death. Studies conducted in sub-Saharan Africa and HICs have shown similar outcomes [29, 30]. Regulations and education on alcohol and illicit drug use, and their acute and chronic effects should be implemented or reinforced on this matter, especially in Sub-Saharan Africa.

Limitations of this study include that it was conducted at ED-MNH, a tertiary care facility and national hospital. Therefore, the results might not be generalizable to other facilities in Tanzania. We tested illicit drugs using a multi drug panel test which could only give the results of five drugs. We may have missed additional illicit drugs that were not included in our drug screen. Finally, we did not measure injury severity directly, but instead used the need for surgical intervention, return to baseline, and death as proxy measures for injury severity.

## Conclusion

The prevalence of alcohol and illicit drug use is very high in patients presenting to the ED-MNH with injury. Most of the patients were young males and the majority of patients who had used drugs or alcohol prior to their injury had not returned to baseline functional level at 30 days post injury. Further studies are needed to generalise the results in Tanzania. Public health initiatives to decrease drinking and driving should be implemented.

## Additional file

Additional file 1: **Table S1.** Relative risks of major surgery return to the baseline, and death among injured patients tested positive for alcohol/illicit drugs (DOC 267 kb)

## Abbreviations

%ED: Emergency Department
95% CI: 95% confidence interval
HIC: High-income countries
IQR: Interquartile Range
LMIC: Low- and Middle-Income Countries
MNH: Muhimbili National Hospital
RR: Relative risk
RTI: Road traffic injuries
WHO: World Health Organization
