# Supplementary material for: The prevalence of alcohol and illicit drug use among injured patients presenting to the emergency department of a national hospital in Tanzania: a prospective cohort study
Source: BMC Emerg Med. 2019 Jan 24;19:15. doi: 10.1186/s12873-019-0222-9 (PMC6346498; doi:10.1186/s12873-019-0222-9)
Supplement: Supplementary file 1 — Table S1. Relative risks of major surgery return to the baseline, and death among injured patients tested positive for alcohol/illicit drugs (DOC 267 kb) [file 12873_2019_222_MOESM1_ESM.doc]

**Supplementary material**

**Additional Table 1.** Relative risks of major surgery return to the baseline, and death among injured patients tested positive for alcohol/illicit drugs

| a. Risk of major surgery for patients who tested positive for alcohol versus those who tested negative for alcohol  RR: 1.42, 95% CI:1.09 to 1.85   |  | Major surgery | |  | | --- | --- | --- | --- | | Yes | No | | Positive alcohol | 49 | 18 | 67 | | Negative alcohol | 39 | 37 | 76 | | Total | 88 | 55 | 143 |   b. Risk of major surgery for patients tested positive for illicit drugs vs those who tested negative for illicit drugs  RR: 1.59, 95% CI: 1.23 to 2.06   |  | Major surgery | | Total | | --- | --- | --- | --- | | Yes | No | | Positive illicit drug | 36 | 8 | 44 | | Negative illicit drug | 40 | 38 | 78 | | Total | 76 | 46 | 122 |   c. Risk of major surgery for patients tested positive for multiple substances ingestion VS single substance ingestion  RR: 0.87, 95% CI: 0.73   |  | Major surgery | | Total | | --- | --- | --- | --- | | Yes | No | | Multiple substance ingestion | 38 | 15 | 53 | | Single substance ingestion | 50 | 8 | 58 | | Total | 88 | 23 | 111 |   d. Risk of major surgery for patients tested positive for Isolated alcohol VS Isolated drugs  RR: 0.93, 95% CI: 0.64   |  | Major surgery | | Total | | --- | --- | --- | --- | |  |  | | Isolated alcohol | 26 | 12 | 38 | | Isolated drugs | 11 | 4 | 15 | | Total | 37 | 16 | 53 |   e. Risk of not returned at baseline for patients who tested positive for alcohol versus those who tested negative for alcohol  RR: 1.19, 95%CI:0.88   |  | Not return at the baseline | |  | | --- | --- | --- | --- | | Yes | No | | Positive alcohol | 40 | 27 | 67 | | Negative alcohol | 38 | 38 | 76 | | Total | 78 | 65 | 143 |   6f. Risk of not returned at baseline for patients tested positive for illicit drugs vs. those who tested negative for illicit drugs  RR: 1.33, 95% CI: 0.97 to 1.83   |  | Not returned at baseline | | Total | | --- | --- | --- | --- | | Yes | No | | Positive illicit drug | 28 | 16 | 44 | | Negative illicit drug | 38 | 40 | 78 | | Total | 66 | 66 | 122 | | g. Risk of not returned at baseline for patients who tested positive for Multiple substances ingestion VS single substance ingestion  RR: 1.12, 95% CI: 0.81 to 1.56   |  | Not returned at base line | | Total | | --- | --- | --- | --- | | Yes | No | | Multiple substances abuse used | 37 | 25 | 58 | | Single substance abuse used | 28 | 25 | 53 | | Total | 65 | 50 | 111 |   h. Risk of not returned at baseline for patients who tested positive for Isolated alcohol VS Isolated illicit drugs  RR: 1.13, 95% CI:0.66 to 1.94   |  | Not returned at the base line | | Total | | --- | --- | --- | --- | | Yes | No | | Isolated alcohol | 23 | 15 | 38 | | Isolated illicit drugs | 8 | 7 | 15 | | Total | 31 | 22 | 53 |   i. Risk of death for patients who tested positive for alcohol versus those who tested negative for alcohol  RR: 1.70, 95% CI: 0.50   |  | Died | |  | | --- | --- | --- | --- | | Yes | No | | Positive alcohol | 6 | 61 | 67 | | Negative alcohol | 4 | 72 | 76 | | Total | 10 | 133 | 143 |   j. Risk of death for patients tested positive for illicit drugs vs. those who tested negative for illicit drugs  RR: 3.54, 95% CI: 0.67   |  | Died | | Total | | --- | --- | --- | --- | | Yes | No | | Positive illicit drug | 4 | 40 | 44 | | Negative illicit drug | 2 | 76 | 78 | | Total | 6 | 116 | 122 |   k. Risk of death for patients tested positive for multiple substances ingestion VS single substance ingestion  RR: 1.82, 95% CI: 0.34   |  | Died | | Total | | --- | --- | --- | --- | | Yes | No | | Multiple substances ingestion | 4 | 54 | 58 | | Single substance ingestion | 2 | 51 | 53 | | Total | 6 | 105 | 111 |   l. Risk of death for patients who tested positive for Isolated alcohol VS Isolated drugs  RR: 0.39, 95% CI: 0.026 to 5.91   |  | Died | | Total | | --- | --- | --- | --- | |  |  | | Isolated alcohol | 1 | 37 | 38 | | Isolated drug | 1 | 14 | 15 | | Total | 2 | 51 | 53 | |
| --- | --- | --- | --- | --- | --- | --- | --- | --- | --- | --- | --- | --- | --- | --- | --- | --- | --- | --- | --- | --- | --- | --- | --- | --- | --- | --- | --- | --- | --- | --- | --- | --- | --- | --- | --- | --- | --- | --- | --- | --- | --- | --- | --- | --- | --- | --- | --- | --- | --- | --- | --- | --- | --- | --- | --- | --- | --- | --- | --- | --- | --- | --- | --- | --- | --- | --- | --- | --- | --- | --- | --- | --- | --- | --- | --- | --- | --- | --- | --- | --- | --- | --- | --- | --- | --- | --- | --- | --- | --- | --- | --- | --- | --- | --- | --- | --- | --- | --- | --- | --- | --- | --- | --- | --- | --- | --- | --- | --- | --- | --- | --- | --- | --- | --- | --- | --- | --- | --- | --- | --- | --- | --- | --- | --- | --- | --- | --- | --- | --- | --- | --- | --- | --- | --- | --- | --- | --- | --- | --- | --- | --- | --- | --- | --- | --- | --- | --- | --- | --- | --- | --- | --- | --- | --- | --- | --- | --- | --- | --- | --- | --- | --- | --- | --- | --- | --- | --- | --- | --- | --- | --- | --- | --- | --- | --- | --- | --- | --- | --- | --- | --- | --- | --- | --- | --- | --- | --- | --- | --- | --- | --- | --- | --- | --- | --- | --- | --- | --- | --- | --- | --- | --- | --- | --- | --- | --- | --- | --- | --- | --- | --- | --- | --- | --- | --- | --- | --- |
